# Antibiotic Resistance of *Campylobacter* Species Isolated From Foods, Animals and Humans in Iran Through One Health Approach: A Systematic Review and Meta‐Analysis

**DOI:** 10.1002/vms3.70746

**Published:** 2025-12-30

**Authors:** Fatemeh Salmani, Sara Mohamadi, Taurai Tasara, Elham Ansarifar, Parisa Sadighara, Tayebeh Zeinali

**Affiliations:** ^1^ Department of Epidemiology and Biostatistics School of Health Geriatric Health Research Center Birjand University of Medical Sciences Birjand Iran; ^2^ Department of Food Hygiene and Quality Control Faculty of Veterinary Medicine Shahrekord University Shahrekord Iran; ^3^ Institute for Food Safety and Hygiene Vetsuisse Faculty University of Zurich Zurich Switzerland; ^4^ Department of Nutrition and Food Hygiene School of Health Social Determinants of Health Research Center Birjand University of Medical Sciences Birjand Iran; ^5^ Department of Environmental Health Food Safety Division School of Public Health Tehran University of Medical Sciences Tehran Iran; ^6^ Department of Nutrition and Food Hygiene School of Health Geriatric Health Research Center Birjand University of Medical Sciences Birjand Iran

**Keywords:** antimicrobial resistance, *Campylobacter*, Iran

## Abstract

In this study, a systematic review and meta‐analysis were conducted to consider antimicrobial resistance (AMR) rates of *Campylobacter* in Iran. A systematic search was conducted in the databases of PubMed, Scopus and Web of Science and reported according to the Preferred Reporting Items for Systematic Reviews and Meta‐Analyses guidelines. Data analysis was done with R software. A number of 58 articles in the current study evaluated AMR in *Campylobacter* species in human, animal and food isolates. Our findings demonstrated that most of the *Campylobacter* spp. isolates in Iran have high resistance proportions to beta‐lactams (including cefixime (0.97), cephalothin (0.84), ceftriaxone (0.76), cephalexin (0.63), ceftazidime (0.53) and carbenicillin (0.38)), fluoroquinolones (including ofloxacin (0.79), nalidixic acid (0.51) and ciprofloxacin (0.52)), sulfonamides like trimethoprim‐sulfamethoxazole (0.68) (potentiated sulfonamides), florfenicol (0.62), tetracycline (0.57) and macrolides (including erythromycin (0.12) and azithromycin (0.17)). Conversely, *Campylobacter* spp. showed low resistance rates to aminoglycosides (including neomycin (0.08), amikacin (0.08), meropenem (0.06), spectinomycin (0.04), gentamicin (0.04) and imipenem (0.02)) and chloramphenicol (0.07). Regarding *Campylobacter coli* and *Campylobacter jejuni* isolates, resistance to erythromycin (0.18–0.09), and gentamycin (0.08–0.04) was higher in *C. coli* than *C. jejuni* isolates, respectively, whereas resistance to ciprofloxacin (0.56) and tetracycline (0.56) was higher in *C. jejuni*. The most prevalent antimicrobial resistance genes (ARGs) in *Campylobacter* spp. were *tetO* (0.73) and *cmeB* (0.48) and *bla*_OXA61_ (0.42). Therefore, the use of strict control systems and a restriction on the use of antibiotics in human, agricultural and animal farming are urgently required to reduce the development and spread of AMR.

## Graphical abstract

Forest plot of pooled prevalence/proportion of erythromycin resistance in *Campylobacter coli* (a) *and C. jejuni* (b). *C. coli* isolates (0.18) had twice resistance to erythromycin than *C. jejuni* isolates (0.09).

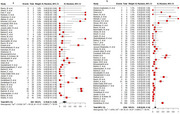

**Abbreviations**
AMR: antimicrobial resistance
ARGs: antimicrobial resistance genes
AST: antimicrobial susceptibility testing
DD: disk diffusion
Fig: figure
MDR: multidrug resistant
MIC: minimum inhibitory concentration
PCR: polymerase chain reaction
PRISMA: Preferred Reporting Items for Systematic Reviews and Meta‐Analyses
SID: Scientific Information Database

## 1 Introduction

*Campylobacter* genus are curved and S‐shaped microaerophilic Gram‐negative bacteria in the family *Campylobacteraceae* (Aminshahidi et al. 2017; Feizabadi et al. 2007; Rahimi et al. 2011). The *Campylobacteraceae* family comprises 18 species, 6 subspecies and 2 biovars. The most significant *Campylobacter* spp. attributed to human illness are thermophilic *Campylobacters* such as *Campylobacter jejuni* and *Campylobacter coli* (Hamidian et al. 2011; Rahimi et al. 2013a). More than 90% of the clinical cases are due to *C. jejuni*, whereas the incidence of *C. coli* is comparatively lesser (approximately 10%) (Azizian et al. 2019; Khoshbakht et al. 2016; Sharifi et al. 2021). The infective dose of this bacterium is exceptionally low; it has been assessed that 500 cells of *C. jejuni* can cause human disease (Ghane et al. 2011).

*Campylobacter* is a commensal of the intestinal tract of an extensive range of food animals, mammals and birds (Bakhshi et al. 2016). Poultry are considered to be the main potential reservoirs of these bacteria (Abbasi et al. 2019a; Khoshbakht et al. 2016; Maktabi et al. 2019). Utilization of contaminated water, unpasteurized milk, insufficiently cooked meat, cross‐contamination of prepared foods and coordination of contact with animals are the most common causes of campylobacteriosis (Rahimi et al. 2013b; Samad et al. 2019). Large‐scale epidemics of human campylobacteriosis are uncommon and are generally associated with the utilization of polluted water or raw milk. Campylobacteriosis is more frequently found in sporadic cases and due to consumption of undercooked chicken (Rahimi et al. 2017). They are one of the foremost common causes of acute diarrhoea, particularly in children under 3 years of age and older adults (Abbasi et al. 2019b). *Campylobacter* spp. strains are claimed to be the cause of 5.4%–10.8% of severe diarrhoea in Iran, which results in the death of 516 children under the age of 5 per year (Aminshahidi et al. 2017; Fani et al. 2019).

The clinical symptoms of campylobacteriosis range from asymptomatic and self‐limiting gastroenteritis to severe inflammatory bloody diarrhoea, abdominal pain and fever (Hamidian et al. 2011). Furthermore, the acute phase of the disease can be attributed to other complications, such as meningitis, myocarditis attack, reactive arthritis, *Guillain‐Barre* syndrome, *Miller Fischer* syndrome, inflammatory bowel syndrome and urinary tract infection (Fani et al. 2019; Maktabi et al. 2019; Sharifi et al. 2021). In immunocompromised patients, the elderly, infants and acute cases that require therapeutic interventions, macrolides (i.e., erythromycin and azithromycin) and fluoroquinolones (i.e., ciprofloxacin) are currently considered the preferred medications for treatment (Azizian et al. 2019; Fani et al. 2019; Khademi and Sahebkar 2020). Tetracyclines and gentamicin are alternative drugs (Khademi and Sahebkar 2020). Additionally, the incidence of human campylobacteriosis is on the rise globally due to the increase of antimicrobial resistance (AMR) in *Campylobacter* spp. (Jonaidi‐Jafari et al. 2016; Rahimi et al. 2017). In 2019, an estimated 1.27 million deaths occurred worldwide that were directly attributed to antimicrobial‐resistant bacteria. Multidrug‐resistant (MDR) *Campylobacter* in livestock, broiler flocks, poultry and red meat (Abbasi et al. 2019b; Abdi‐Hachesoo et al. 2014; Fani et al. 2019; Hamidian et al. 2011; Sharifi et al. 2021) has been reported to date in multiple Iranian studies. Antimicrobial‐resistant microbial strains hold the potential to traverse the food chain, rendering them accessible to humans (Dallal et al. 2010; Fani et al. 2019; Maktabi et al. 2019; Ghane et al. 2011). Therefore, an improved understanding of the prevalence of the AMR in *Campylobacter* is a crucial step in creating efficient methods for reducing the occurrence and transmission of antimicrobial‐resistant *Campylobacter* from food animal production and their immediate environment to humans.

*Campylobacter* is listed among the significant etiological agents associated with gastroenteritis in Iran (Rahimi et al. 2013b), but a comprehensive national report on the AMR patterns of *Campylobacter* spp. remains unavailable, primarily due to the absence of sustainable surveillance systems for foodborne zoonotic pathogens and their resistance to antimicrobials. To address this gap, the present systematic review and meta‐analysis was undertaken to assess the prevalence of AMR in *Campylobacter* spp. isolated from humans, animals and food sources in Iran, as well as to examine the distribution of antibiotic resistance genes (ARGs) from an One Health perspective. Although this study shares certain thematic overlaps with the work of Khademi and Sahebkar (2020), it incorporates a broader range of investigations and thus provides a more extensive evidence base. The findings of this review are expected to generate critical data that can inform optimal antimicrobial therapy for infections caused by *Campylobacter* spp. Furthermore, the results will be instrumental in shaping national strategies for the prevention and control of *Campylobacter* infections, whereas simultaneously highlighting key research gaps that warrant future exploration.

## 2 Methods and Materials

### 2.1 Search Strategy

This study was performed as a systematic review of *Campylobacter* prevalence in Iran (Ansarifar et al. 2023). To identify the qualified investigations, scientific digital databases such as PubMed, Web of Science, Scopus, Google Scholar and Scientific Information Database (SID) were searched with the following keywords: *C. coli* or *C. jejuni* combined with the following terms: ‘Food’, ‘Animal’, ‘Chicken’, ‘Poultry’, ‘Meat’, ‘Beef’, ‘Lamb’, ‘Fish’, ‘Milk’, ‘Dairy’, ‘Egg’, ‘Sheep’, ‘Goat’, ‘Avian’, ‘Cow’, ‘Cattle’, ‘Human’, ‘Faeces’, ‘Diarrhoea’, ‘Gastroenteritis’ and ‘Iran’ and reported according to Preferred Reporting Items for Systematic Reviews and Meta‐Analyses (PRISMA) guidelines.

### 2.2 Criteria for Inclusion and Exclusion

The consideration standards included published or in‐press original articles with a cross‐sectional design that reported AMR in *Campylobacter* isolates and studies with a characterized test size. There was no restriction on the antimicrobial susceptibility testing (AST) method. To select eligible articles, the titles and abstracts of the acquired articles were reviewed. In some circumstances full texts were evaluated. The exclusion criteria were as follows: (1) duplicate articles from different databases, (2) articles that did not follow culture or molecular methods for *Campylobacter* isolation, (3) articles with unclear or incomprehensible text and analysis that did not show the accurate results of AMR in Campylobacter, (4) articles that did not report the specific sample size and number/percent of *Campylobacter*, (5) positive sample reviews; letters or editorial articles without original data and (6) articles did not report the AMR of *Campylobacter spp*.

### 2.3 Data Extraction

Data extraction was carried out in Microsoft Excel. Articles that met 6 of 10 criteria of the Joanna Briggs checklist (Munn et al. 2014) (i.e., 60% of the quality score) were included in the analysis. The following information was obtained: author's name, publication year, study year, study design, number of isolates and AMR of *Campylobacter* spp., *C. jejuni*, *C. coli* and AST method.

### 2.4 Risk of Bias Assessment

The quality evaluation of the eligible articles was performed in accordance with the risk of bias assessment table of the Joanna Briggs Institute (Munn et al. 2014).

### 2.5 Statistical Analysis

The analysis of the data was carried out using R 4.2 software with metafor packages to determine the pooled prevalence and 95% confidence interval of antibiotic resistance of *Campylobacter* species by random effects model. Statistical heterogeneity among studies was evaluated by computing *I* ^2^, Cochran's *Q*. 25%, 50% and 75% of *I* ^2^ values were classified as low, medium and high heterogeneity, respectively.

## 3 Results

**FIGURE 1:**
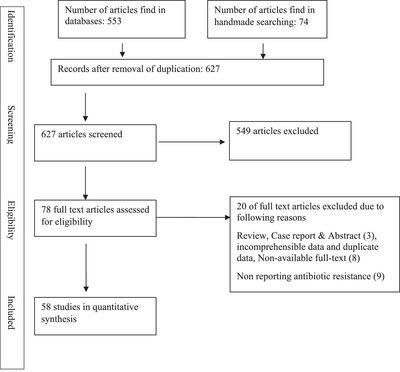
Flowchart of article selection according to Preferred Reporting Items for Systematic Reviews and Meta‐Analyses (PRISMA).

A total number of 627 articles were searched and screened. A number of 58 articles in the current study from 2004 to 2025 evaluated AMR in *Campylobacter* species, including *C. jejuni* and *C. coli*. Reviews, case reports, abstracts, confused text/incomprehensible, duplicates and non‐available full‐text articles were excluded. All the studies that isolated *Campylobacter* from human, animals or foods and reported their AMR to some antibiotics were included. Most of the articles investigated AMR through the disk diffusion (DD) method. AMR data that were reported in two studies just one time extracted and inserted in the meta‐analysis. Figure 1 shows the searched diagram of articles. Table 1 shows the characteristics of the included studies. A list of excluded articles can be available upon request.

**TABLE 1:** Characteristics of the included studies.

	Reference	Study year	Sample source	Sample type	*Campylobacter* species (isolates number)	Antibiotics tested	Method	Quality assessment
1	Abbasi et al. (2019a)	2015	Human	Diarrhoea	*Campylobacter coli* (5)	Ampicillin, ciprofloxacin, erythromycin, gentamicin, tetracycline	Disk diffusion test (DD)	10
2	Abbasi et al. (2019b)	2015	Human	Diarrhoea	*Campylobacter jejuni* (76)	Ampicillin, ciprofloxacin, erythromycin, gentamicin, tetracycline	DD, minimum inhibitory concentration (MIC) by antibiotic gradient testing, polymerase chain reaction (PCR)	10
3	Abdi‐Hachesoo et al. (2014)	2009	Food	White meat	*Campylobacter jejuni* (43) and *coli* (40)	Tetracycline	PCR	10
4	Ahmadi et al. (2023)	2022	Food	Milk	*Campylobacter jejuni* (6) and *coli* (10)	Amoxicillin/Clavulanic acid, azithromycin, ceftriaxone, chloramphenicol doxycycline, erythromycin, nalidixic acid, tetracycline	DD	10
5	Aminshahidi et al. (2017)	2014–2015	Human	Diarrhoea	*Campylobacter jejuni* (7)	Ampicillin, azithromycin, ciprofloxacin, gentamicin, meropenem, nalidixic acid, tetracycline	DD	9
6	Ashrafganjooyi and Saeid‐Adeli (2016)	2008–2010	Animal (poultry)	Cecal content	*Campylobacter jejuni* (190)	Ampicillin, tetracycline, trimethoprim‐sulfamethoxazole	DD	10
7	Azizian et al. (2019)	2015–2016	Animal (poultry)	Cecal content	*Campylobacter jejuni* (50) and *coli* (8)	Amoxicillin, ampicillin, ciprofloxacin, erythromycin, gentamicin, tetracycline, meropenem	DD and agar dilution method	10
8	Basiri et al. (2016)	2014–2015	Animal (poultry)	Faeces	*Campylobacter jejuni* (78) and *coli* (20)	Amoxicillin, ampicillin, chloramphenicol, ciprofloxacin, enrofloxacin, erythromycin, gentamicin, nalidixic acid, neomycin, spectinomycin, streptomycin, tetracycline	DD	10
9	Bakhshi et al. (2016)	2012	Food	White meat	*Campylobacter coli* (39)	Amoxicillin, ampicillin, chloramphenicol, ciprofloxacin, erythromycin, gentamicin, nalidixic acid, streptomycin, tetracycline, trimethoprim‐sulfamethoxazole	DD	10
10	Basirisaehi et al. (2007)	2006	Animal (cows, horse, poultry)	Faeces	*Campylobacter jejuni* (15), *coli* (10) and *lari* (12)	Ampicillin, cefotaxime, cephalexin, chloramphenicol, ciprofloxacin, erythromycin, gentamicin, tetracycline, trimethoprim‐sulfamethoxazole	DD, E‐test	8
11	Dabiri et al. (2016)	2011–2012	Food	Red and white meat	*Campylobacter jejuni* (93) and *coli* (28)	Amoxicillin, ampicillin, chloramphenicol, ciprofloxacin, colistin, erythromycin, gentamicin, nalidixic acid, neomycin, spectinomycin, streptomycin, tetracycline	DD	10
12	Emami et al. (2023)		Food	Mushrooms	*Campylobacter jejuni* (32) and *coli* (11)	Amoxicillin + clavulanic acid, ampicillin azithromycin, chloramphenicol, ciprofloxacin, clindamycin, erythromycine, gentamicin, nalidixic acid, tetracycline	DD	10
13	Fani et al. (2019)	2016	Food	White meat	*Campylobacter jejuni* (24) and *coli* (2)	Amikacin, ampicillin, azithromycin, cefepime, cefixime, ceftriaxone, cefuroxime, cephalotin, chloramphenicol, ciprofloxacin, erythromycin, gentamicin, meropenem, nalidixic acid, tetracycline, trimethoprim‐sulfamethoxazole	DD	10
14	Feizabadi et al. (2007)	2004–2005	Human	Diarrhoea	*Campylobacter jejuni* (29) and *coli* (5)	Ampicillin, carbenicillin, cefotaxime, ceftazidime, cephalexin, cephalotin, chloramphenicol, ciprofloxacin, colistin, erythromycin, gentamicin, imipenem, nalidixic acid, neomycin, ofloxacin, streptomycin, tetracycline	DD	10
15	Ghane et al. (2011)	2010	Animal (poultry and cattle)	Faeces	*Campylobacter jejuni* (27), *coli* (18) and *lari* (20)	Ampicillin, cefotaxime, cephalexin, chloramphenicol, ciprofloxacin, erythromycin, gentamicin, tetracycline	DD, E‐test	9
16	Ghane et al. (2010)	2010	Animal (cow, horse, sheep, poultry), environment (sewage, and river)	Faeces	*Campylobacter jejuni* (16), *coli* (9) and *lari* (7)	Amikacin, amoxicillin, amoxicillin/clavulanic acid, ampicillin, ceftriaxone, cephalexin, chloramphenicol, ciprofloxacin, erythromycin, gentamicin, penicillin, tetracycline, tobramycin, vancomycin	DD, MIC	10
17	Ghorbanalizadgan et al. (2019)	2018	Human	Diarrhoea	*Campylobacter jejuni* (31) and *coli* (2)	Ampicillin, chloramphenicol, ciprofloxacin, colistin, erythromycin, gentamicin, nalidixic acid, tetracycline, trimethoprim‐sulfamethoxazole	DD	10
18	Gilani et al. (2023)	2021–2022	Animal	Faeces	*Campylobacter jejuni* (3) and *coli* (19)	Amoxicillin–clavulanic acid, ampicillin, ciprofloxacin, erythromycin, gentamicin, nalidixic acid, tetracycline	DD	10
19	Hadiyan, Momtaz and Shakerian (2022)	2022	Food	White meat	*Campylobacter jejuni* (54) and *coli* (26)	Amoxicillin, ampicillin, azithromycin, chloramphenicol, ciprofloxacin, clindamycin erythromycin, gentamicin, nalidixic acid, tetracycline	DD	10
20	Hamidian et al. (2011)	2008–2009	Human	Diarrhoea	*Campylobacter jejuni* (34) and *coli* (12)	Ampicillin, carbenicillin, cefotaxime, ceftazidime, chloramphenicol, ciprofloxacin, colistin, erythromycin, gentamicin, imipenem, nalidixic acid, neomycin, ofloxacin, streptomycin, tetracycline	DD	9
21	Irajian et al. (2008)	2007	Human	Diarrhoea	*Campylobacter jejuni* (38)	Ciprofloxacin, erythromycin, gentamicin, tetracycline, trimethoprim‐sulfamethoxazole	DD	10
22	Jamali et al. (2015)	2008–2010	Animal (poultry)	Cecal content	*Campylobacter jejuni* (138) and *coli* (23)	Amoxicillin, ampicillin, chloramphenicol, ciprofloxacin, colistin, erythromycin, gentamicin, nalidixic acid, neomycin, streptomycin, tetracycline	DD	10
23	Jazayeri Moghadas et al. (2008)	2007	Human	Diarrhoea	*Campylobacter jejuni* (27)	Ciprofloxacin, erythromycin, gentamicin, trimethoprim‐sulfamethoxazole, tetracycline	DD	10
24	Jonaidi‐Jafari et al. (2016)	2014–2015	Food	Eggshell and egg content	*Campylobacter jejuni* (28) and *coli* (6)	Amoxicillin, ampicillin, chloramphenicol, ciprofloxacin, enrofloxacin, erythromycin, gentamicin, nalidixic acid, streptomycin, tetracycline	DD	10
25	Khoshbakht et al. (2016)	2011–2013	Animal (cattle, sheep)	Faeces	*Campylobacter jejuni* (48) and *coli* (17)	Ampicillin, cefotaxime, cephalotin, chloramphenicol, ciprofloxacin, colistin, enrofloxacin, erythromycin, gentamicin, nalidixic acid, neomycin, tetracycline, tylosin	DD	10
26	Khosravi et al. (2011)	2007–2008	Human	Diarrhoea	*Campylobacter jejuni* (9) and *coli* (5)	Ampicillin, cefotaxime, ceftazidime, cephalothin, ciprofloxacin, erythromycin, gentamycin, nalidixic acid, oxacillin, tetracycline, trimethoprim‐sulfamethoxazole	DD, E‐test	10
27	Maktabi et al. (2019)	2016	Food	Red and white meat	*Campylobacter jejuni* (26) and *coli* (6)	Ampicillin, chloramphenicol, ciprofloxacin, enrofloxacin, erythromycin, gentamicin, streptomycin, tetracycline	DD	10
28	Mirzaie et al. (2011)	2010	Animal (poultry)	Cecal content	*Campylobacter jejuni* (33) and *coli* (19)	Ampicillin, chloramphenicol, ciprofloxacin, erythromycin, gentamicin, neomycin, nalidixic acid, tetracycline	DD	10
29	Moradi and Baserisalehi (2022)	2022	Animal (poultry, cow)	Faeces	*Campylobacter jejuni* (9) and *coli* (1)	Ampicillin, cefotaxime, ceftriaxone, ciprofloxacin, erythromycin, gentamicin	DD	10
30	Mostafavi and Neyriz‐Naghadehi (2023)	2018	Food	Milk	*Campylobacter jejuni* (11) and *coli* (2)	Ampicillin, ceftriaxone, chloramphenicol, ciprofloxacin, gentamicin, nitrofurantoin, tetracycline, trimethoprim‐sulphamethoxazole	DD	10
31	Mousavinafchi et al. (2023)	2020	Food	White meat	*Campylobacter jejuni* (41) and *coli* (14)	Ciprofloxacin, erythromycin, gentamicin, nalidixic acid, tetracycline	MIC	10
32	Raeisi et al. (2017)	2014–2015	Food	Raw milk, white meat, red meat	*Campylobacter jejuni* (79) and *coli* (41)	Amikacin, amoxicillin, ampicillin, ciprofloxacin, enrofloxacin, erythromycin, gentamicin, nalidixic acid, streptomycin, tetracycline	DD	10
33	Rahimi et al. (2010a)	2008–2009	Food	Red meat	*Campylobacter jejuni* (42) and *coli* (8)	Amoxicillin, ampicillin, chloramphenicol, ciprofloxacin, enrofloxacin, erythromycin, gentamicin, nalidixic acid, streptomycin, tetracycline	DD	10
34	Rahimi et al. (2010b)	2007–2008	Food	Poultry carcass	*Campylobacter jejuni* (177) and *coli* (21)	Amoxicillin, ampicillin, chloramphenicol, ciprofloxacin, enrofloxacin, erythromycin, gentamicin, nalidixic acid, streptomycin, tetracycline	DD	10
35	Rahimi and Ameri (2011)	2009–2010	Food	White meat	*Campylobacter jejuni* (172) and *coli* (15)	Amoxicillin, ampicillin, chloramphenicol, ciprofloxacin, enrofloxacin, erythromycin, gentamicin, nalidixic acid, streptomycin, tetracycline	DD	10
36	Rahimi et al. (2011)	2009–2010	Food	White meat	*Campylobacter jejuni* (46) and *coli* (6)	Amoxicillin, ampicillin, chloramphenicol, ciprofloxacin, enrofloxacin, erythromycin, gentamicin, nalidixic acid, streptomycin, tetracycline	DD	10
37	Rahimi et al. (2013a)	2009–2010	Food	Red meat	*Campylobacter jejuni* (24) and *coli* (7)	Amoxicillin, ampicillin, chloramphenicol, ciprofloxacin, enrofloxacin, erythromycin, gentamicin, nalidixic acid, streptomycin, tetracycline	DD	10
38	Rahimi et al. (2013b)	2011	Food	Red and white meat	*Campylobacter jejuni* (193) and *coli* (20)	Amoxicillin, ampicillin, chloramphenicol, ciprofloxacin, enrofloxacin, erythromycin, gentamicin, nalidixic acid, streptomycin, tetracycline	DD	10
39	Rahimi et al. (2017)	2014–2015	Animal (cattle, sheep, goat, camel)	Faeces	*Campylobacter jejuni* (22) and *coli* (6)	Amoxicillin, ampicillin, chloramphenicol, ciprofloxacin, enrofloxacin, erythromycin, gentamicin, nalidixic acid, streptomycin, tetracycline	DD	10
40	Rahimi et al. (2024)	2020–2021	Food	Red meat	*Campylobacter jejuni* (33) and *coli* (36)	Amoxicillin, ampicillin, azithromycin, chloramphenicol, ciprofloxacin, clindamycin erythromycin, gentamicin, nalidixic acid, tetracycline	DD	10
41	Rastyani et al. (2015)	2013–2014	Human	Diarrhoea	*Campylobacter jejuni* (6) and *coli* (3)	Amikacin, chloramphenicol, ciprofloxacin, erythromycin, gentamicin, meropenem, tetracycline	DD	9
42	Saadatmand et al. (2017)	2016	Food	Organ meat (poultry liver)	*Campylobacter jejuni* (53) and *coli* (19)	Amoxicillin, ampicillin, chloramphenicol, ciprofloxacin, colistin, erythromycin, gentamicin, streptomycin, tetracycline, trimethoprim‐sulfamethoxazole	DD	10
43	Sabzmeydani et al. (2020)	2018–2019	Food	Poultry eggshell	*Campylobacter jejuni* (45) and *coli* (39)	Amoxicillin, ampicillin, cephalothin, chloramphenicol, ciprofloxacin, colistin, enrofloxacin, erythromycin, gentamicin, nalidixic acid, neomycin, streptomycin, tetracycline	DD	
44	Sadeghi et al. (2020)	2019–2020	Human	Diarrhoea	*Campylobacter* spp. (1), *jejuni* (24), *coli* (2) and *lari* (1)	Ampicillin, ciprofloxacin, clindamycin, erythromycin, gentamicin, nalidixic acid, tetracycline	Agar diffusion test, DD, E‐test, PCR	10
45	Sadeghi et al. (2022)		Food	White meat	*Campylobacter* spp. (9), *jejuni* (23), *coli* (1) and *lari* (2)	Ampicillin, ciprofloxacin, clindamycin, erythromycin, gentamicin, nalidixic acid, tetracycline	Agar diffusion test, DD, E‐test, PCR	10
46	Salehi et al. (2014)	2011–2013	Human	Diarrhoea	*Campylobacter jejuni* (19)	Cephalotin, erythromycin, nalidixic acid	DD	10
47	Shafiei et al. (2020)	2018–2019	Food and animal (cattle, sheep, goat)	Meat, liver, kidney, heart and contents of rectum	*Campylobacter jejuni* (69) and *coli* (45)	Amikacin, amoxicillin, ampicillin, cefazolin, chloramphenicol, ciprofloxacin, erythromycin, gentamicin, imipenem, meropenem, nalidixic acid, norfloxacin, streptomycin, tetracycline	DD	9
48	Shahrokhabad et al. (2011)	2010	Food	White meat and organ meat	*Campylobacter jejuni* (19) and *coli* (12)	Ciprofloxacin, erythromycin, gentamicin, nalidixic acid, trimethoprim‐sulfamethoxazole	DD	10
49	Shakerian et al. (2012)	2006–2008	Food	Red meat	*Campylobacter jejuni* (13) and *coli* (4)	Amoxicillin, ampicillin, chloramphenicol, ciprofloxacin, enrofloxacin, erythromycin, gentamicin, nalidixic acid, streptomycin, tetracycline	DD	10
50	Shakerian (2016)	2014	Food	Vegetable (mushroom)	*Campylobacter jejuni* (2) and *coli* (13)	Amoxicillin, ampicillin, chloramphenicol, ciprofloxacin, enrofloxacin, erythromycin, gentamicin, nalidixic acid, streptomycin, tetracycline	DD	10
51	Sharifi et al. (2021)	2019–2020	Human	Diarrhoea	*Campylobacter jejuni* (18) and *coli* (2)	Chloramphenicol, ciprofloxacin, erythromycin, gentamicin, minocycline, nalidixic acid, tetracycline	DD, MIC by broth microdilution method, PCR (*tet O, gyrA*)	10
52	Shirazi et al. (2013)	2011	Human	Diarrhoea	*Campylobacter jejuni* (9)	Ciprofloxacin, erythromycin, gentamicin, tetracycline, trimethoprim‐sulfamethoxazole	DD	10
53	Dallal et al. (2010)	2006–2007	Food	Red and white meat	*Campylobacter jejuni* (70) and *coli* (22)	Amoxicillin, ampicillin, chloramphenicol, ciprofloxacin, colistin, erythromycin, gentamicin, nalidixic acid, neomycin, spectinomycin, streptomycin, tetracycline	DD	10
54	Taremi et al. (2006)	2004	Food	Red and white meat	*Campylobacter* spp. (80)	Amoxicillin, chloramphenicol, ciprofloxacin, erythromycin, gentamicin, nalidixic acid, streptomycin, tetracycline	DD	10
55	Zamani Moghaddam et al. (2012)	2010–2011	Environment—pigeon	Faeces	*Campylobacter jejuni* (1)	Ampicillin, ciprofloxacin, doxycycline, enrofloxacin, erythromycin, florfenicol, oxytetracycline, trimethoprim‐sulfamethoxazole	DD	10
56	Zamani Moghadam et al. (2013)	2011	Environment—lovebird	Faeces	*Campylobacter* spp. (2)	Ampicillin, ciprofloxacin, doxycycline, enrofloxacin, erythromycin, florfenicol, oxytetracycline, trimethoprim‐sulfamethoxazole	DD	10
57	Zendehbad et al. (2013)	2012	Food	White meat	*Campylobacter jejuni* (122) and *coli* (27)	Amoxicillin, ampicillin, chloramphenicol, ciprofloxacin, colistin, enrofloxacin, erythromycin, gentamicin, nalidixic acid, neomycin, spectinomycin, streptomycin, tetracycline	DD	10
58	Zendehbad et al. (2015)	2013	Food	White meat	*Campylobacter jejuni* (200) and *coli* (27)	Amoxicillin, ampicillin, chloramphenicol, ciprofloxacin, enrofloxacin, erythromycin, gentamicin, nalidixic acid, neomycin, tetracycline	DD	10

### 3.1 Pooled Prevalence of AMR in *Campylobacter* spp

Table 2 shows the AMR proportions among *Campylobacter* spp. in human, food and animal subgroups. A total of 58 studies reported the antibiotic resistance in human (faeces), animal faeces and food, including meat, milk, eggs or vegetables. As displayed, the most frequent resistance among *Campylobacter* spp. was to cefixime (0.97), cephalothin (0.84), ofloxacin (0.79), ceftriaxone (0.76), trimethoprim‐sulfamethoxazole (0.68), cephalexin (0.63) and florfenicol (0.62). Resistance to tetracycline (0.57), doxycycline (0.54), ceftazidime (0.53), ciprofloxacin (0.52), nalidixic acid (0.51), amoxicillin–clavulanic acid (0.34), clindamycin (0.34), ampicillin (0.29), azithromycin (0.17) and erythromycin (0.12) was high. The lowest resistance frequency was detected for imipenem (0.02), spectinomycin (0.04), gentamicin (0.04), meropenem (0.06) and chloramphenicol (0.07). *Campylobacter* spp. isolates had different resistance proportions in different sectors (Table 2). In most of the investigated antibiotics (nine types), resistance was higher in human isolates than other ones. Food isolates had higher resistance to some antibiotics, including doxycycline, neomycin, imipenem, azithromycin, colistin, ciprofloxacin, trimethoprim‐sulfamethoxazole and tetracycline.

**TABLE 2:** The subgroup analysis of antimicrobial resistance (AMR) rates among *Campylobacter* spp.

World Health Organization's categorization of antibiotics (WHO 2024)	Antibiotic	Study number	Resistance proportions	Lower bound	Upper bound	*I* ^2^	Sample's type	No. study	Resistance proportions	Lower bound	Upper bound	*I* ^2^
Authorized for use in humans only	Carbenicillin	2	0.38	0.28	0.49	0	Human	2	0.38	0.28	0.49	0
	Imipenem	3	0.02	0.01	0.05	0	Human	2	0.01	0.00	0.08	0
							Food	1	0.02	0.00	0.07	—
	Meropenem	6	0.06	0.01	0.25	76	Animal	1	0	0	0.05	—
							Human	3	0.11	0.01	0.68	82
							Food	2	0.07	0.02	0.22	28
Highest priority critically important antimicrobial	Cefixime	2	0.97	0.82	1	0	Animal	1	1	0.69	1	—
							Food	1	1	0.87	1	—
	Cefotaxime	6	0.32	0.16	0.5	92	Animal	3	0.25	0.08	0.56	95
							Human	3	0.4	0.15	0.73	85
	Ceftazidime	3	0.53	0.43	0.63	22	Human	3	0.53	0.43	0.63	22
	Ceftriaxone	5	0.76	0.53	0.90	67	Animal	2	0.83	0.48	0.96	40
							Food	3	0.72	0.36	0.92	79
	Ciprofloxacin	53	0.52	0.45	0.59	85.6	Animal	13	0.43	0.19	0.71	92
							Human	13	0.45	0.28	0.63	82
							Food	27	0.51	0.40	0.61	93
	Enrofloxacin	18	0.23	0.18	0.29	84. 3	Animal	4	0.31	0.14	0.54	75.1
							Food	14	0.21	0.15	0.29	90
	Nalidixic acid	39	0.51	0.43	0.59	87.9	Animal	6	0.54	0.36	0.72	92
							Human	8	0.53	0.35	0.70	78
							Food	25	0.48	0.34	0.62	93
	Ofloxacin	2	0.79	0.68	0.86	0	Human	2	0.79	0.68	0.86	0
	Colistin	11	0.23	0.1	0.45	92	Animal	2	0.03	0	0.81	92
							Human	3	0.21	0.01	0.87	95
							Food	6	0.31	0.16	0.50	93
Critically important antimicrobials	Amikacin	3	0.08	0.03	0.18	73	Animal	3	0.08	0.03	0.18	73
	Gentamicin	50	0.04	0.03	0.07	79.5	Animal	10	0.05	0.02	0.11	75
							Human	13	0.09	0.04	0.18	67
							Food	27	0.03	0.01	0.06	85
	Neomycin	12	0.08	0.05	0.14	86	Animal	4	0.06	0.01	0.35	92
							Human	2	0.07	0.02	0.20	31
							Food	6	0.08	0.05	0.14	84
	Azithromycin	5	0.17	0.09	0.29	50	Human	1	0	0	0.41	—
							Food	4	0.18	0.10	0.31	57
	Erythromycin	52	0.12	0.07	0.16	89.5	Animal	12	0.14	0.06	0.31	88
							Human	13	0.23	0.11	0.43	80
							Food	27	0.07	0.03	0.15	92
Highly important antimicrobials	Chloramphenicol	34	0.07	0.04	0.11	86.9	Animal	6	0.08	0.05	0.14	65.3
							Human	5	0.07	0.04	0.13	0
							Food	23	0.07	0.03	0.14	91
	Florfenicol	2	0.62	0.16	0.93	0	Animal	2	0.62	0.16	0.93	0
	Cephalexin	4	0.63	0.32	0.86	88	Animal	3	0.50	0.28	0.71	81
							Human	1	0.91	0.77	0.98	—
	Cephalotin	5	0.84	0.47	0.97	95	Animal	1	0.26	0.20	0.33	—
							Human	2	0.95	0.84	0.99	0
							Food	2	0.87	0.42	0.98	78
	Clindamycin	5	0.34	0.25	0.43	52	Human	2	0.49	0.25	0.74	49
							Food	3	0.29	0.23	0.35	23
	Amoxicillin	26	0.16	0.11	0.23	89.8	Animal	5	0.31	0.14	0.56	88.8
							Human	1	0	0	0.07	—
							Food	20	0.13	0.08	0.22	91
	Ampicillin	46	0.29	0.22	0.37	91	Animal	14	0.32	0.17	0.51	94
							Human	8	0.51	0.23	0.78	89
							Food	24	0.21	0.13	0.31	92
	Amoxicillin–clavulanic acid	3	0.34	0.19	0.52	68	Animal	1	0.52	0.32	0.71	—
							Food	2	0.26	0.18	0.35	0
	Trimethoprim‐sulfamethoxazole	14	0.68	0.51	0.82	87	Animal	5	0.62	0.21	0.91	93
							Human	4	0.49	0.39	0.58	0
							Food	5	0.81	0.74	0.86	0
	Doxycycline	3	0.54	0.32	0.74	0	Animal	2	0.42	0.05	0.91	31
							Food	1	0.56	0.30	0.80	—
	Tetracycline	53	0.57	0.05	0.64	89.5	Animal	13	0.45	0.25	0.67	94
							Human	12	0.51	0.36	0.65	75
							Food	28	0.59	0.46	0.70	94
Important antimicrobials	Spectinomycin	5	0.04	0.02	0.06	6	Animal	1	0.05	0.02	0.12	—
							Food	4	0.03	0.01	0.06	11

### 3.2 Pooled Prevalence of Ampicillin, Ciprofloxacin, Erythromycin, Gentamycin and Tetracycline Resistance Proportions in *Campylobacter coli* and *jejuni* Isolates

Ampicillin, ciprofloxacin, erythromycin, gentamycin and tetracycline were among the commonly used antibiotics in the studies, so resistance of *C. jejuni* and *C. coli* to these agents was investigated separately. Ampicillin resistance in *C. coli* isolates (0.3, 95% CI: 0.2–0.42; *I* ^2^: 78.9%) was the same as *C. jejuni* isolates (0.3, 95% CI: 0.21–0.4; *I* ^2^: 89.7%). Ciprofloxacin resistance was higher in *C. jejuni* isolates (0.56, 95% CI: 0.46–0.66; *I* ^2^: 90.1%) than *C. coli* isolates (0.53, 95% CI: 0.43–0.62; *I* ^2^: 71.6%). *C. coli* isolates (0.18, 95% CI: 0.11–0.28; *I* ^2^: 78.5%) had twice the resistance to erythromycin than *C. jejuni* isolates (0.09, 95% CI: 0.06–0.14; *I* ^2^: 85.4%). Moreover, gentamycin resistance in *C. jejuni* (0.04, 95% CI: 0.03–0.07; *I* ^2^: 75.5%) was half of *C. coli* isolates (0.08, 95% CI: 0.05–0.11; *I* ^2^: 22.8%). The resistance rates to tetracycline in *C. jejuni* (0.56, 95% CI: 0.47–0.65; *I* ^2^: 91.1%) were more than in *C. coli* isolates (0.53, 95% CI: 0.43–0.62; *I* ^2^: 71.6%). Figure 2 shows the forest plot of these antimicrobials in *C. coli* and *C. jejuni* isolates.

FIGURE 2Pooled prevalence of ampicillin, ciprofloxacin, erythromycin, gentamycin and tetracycline resistance proportions in *Campylobacter coli* and *jejuni* isolates. (a) Ampicillin resistance in *C. coli*; (b) ampicillin resistance in *C. jejuni*; (c) ciprofloxacin resistance in *C. coli*; (d) ciprofloxacin resistance in *C. jejuni*; (e) erythromycin resistance in *C. coli*; (f) erythromycin resistance in *C. jejuni*; (g) gentamycin resistance in *C. coli*; (h) gentamycin resistance in *C. jejuni*; (i) tetracycline resistance in *C. coli*; (j) tetracycline resistance in *C. jejuni*.

**Figure:**
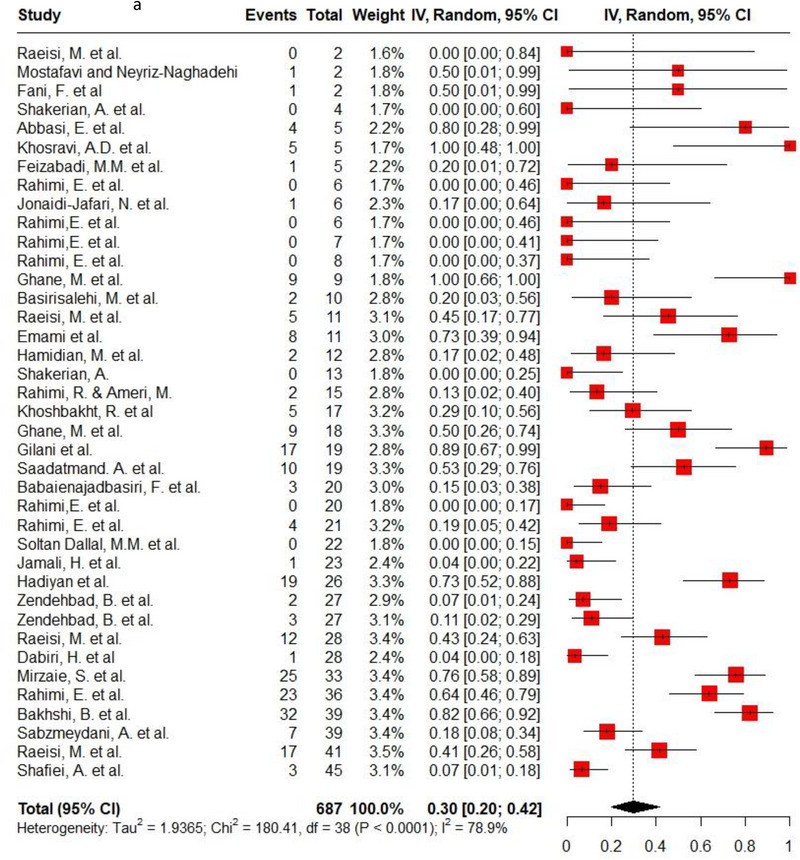

**Figure:**
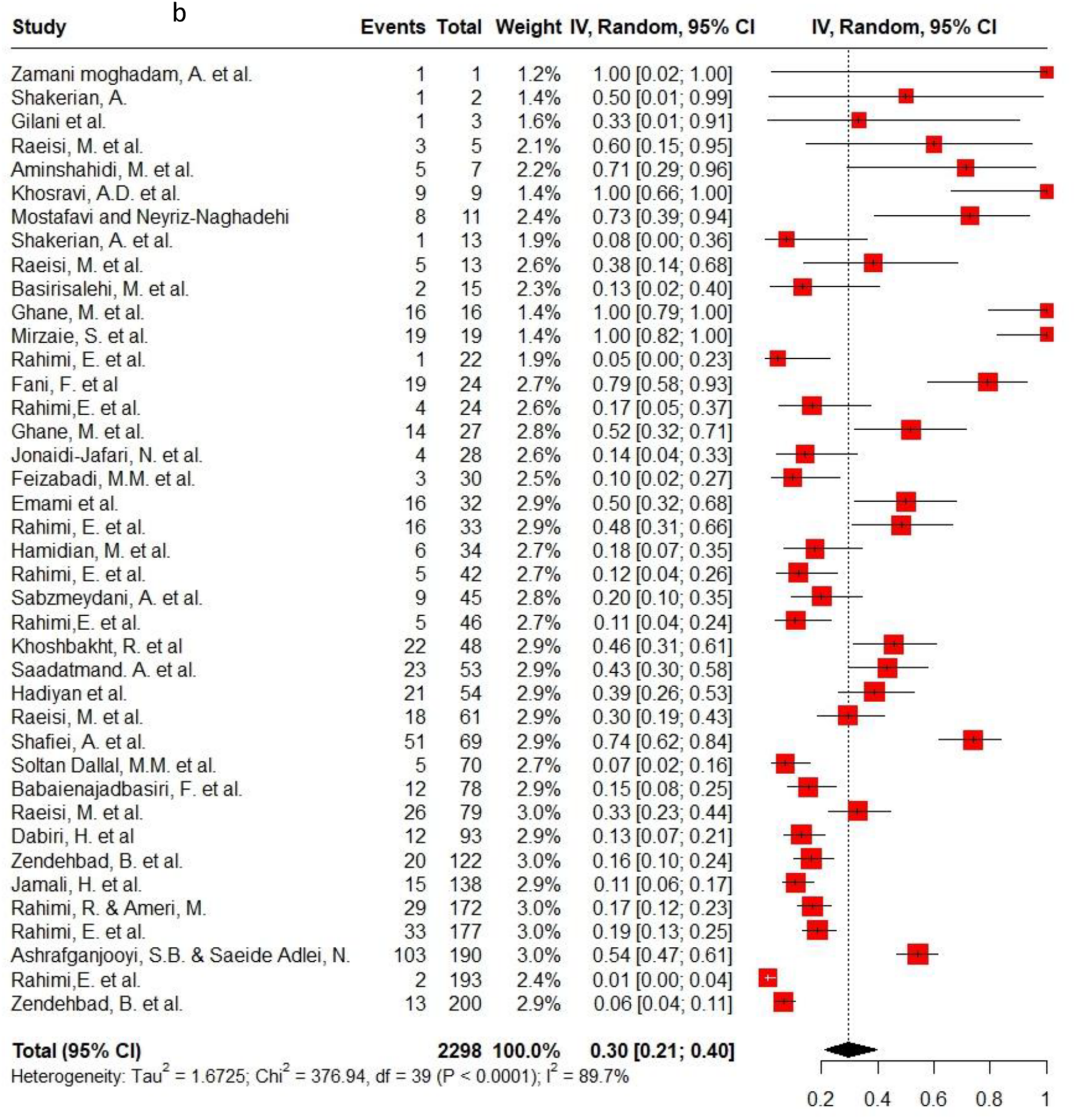

**Figure:**
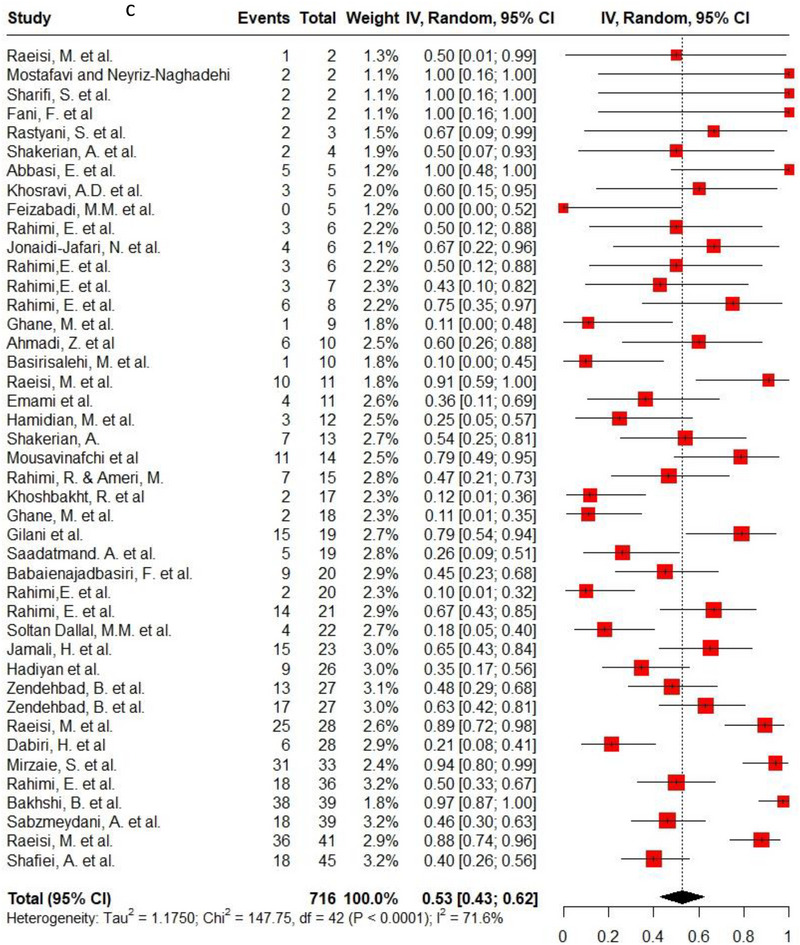

**Figure:**
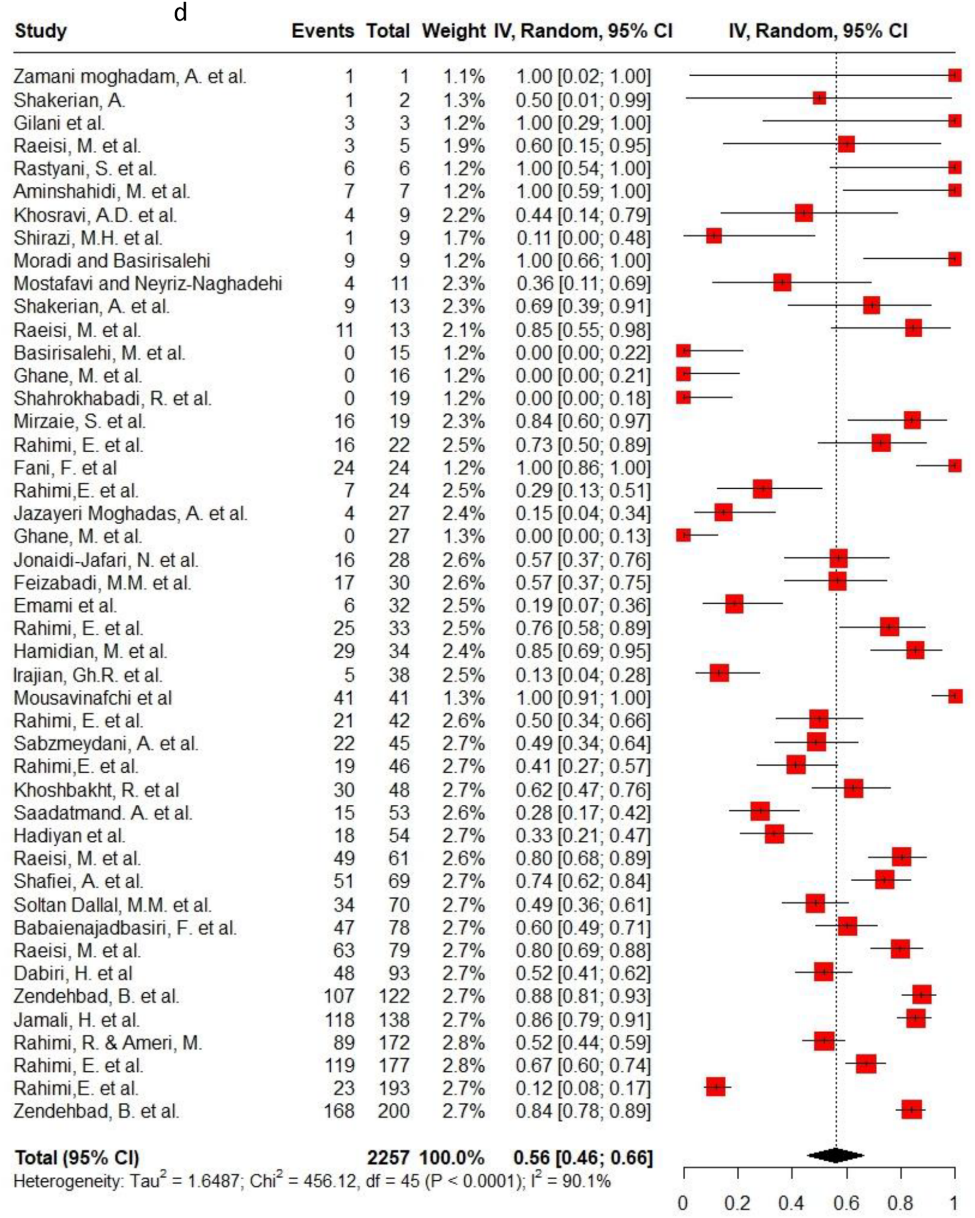

**Figure:**
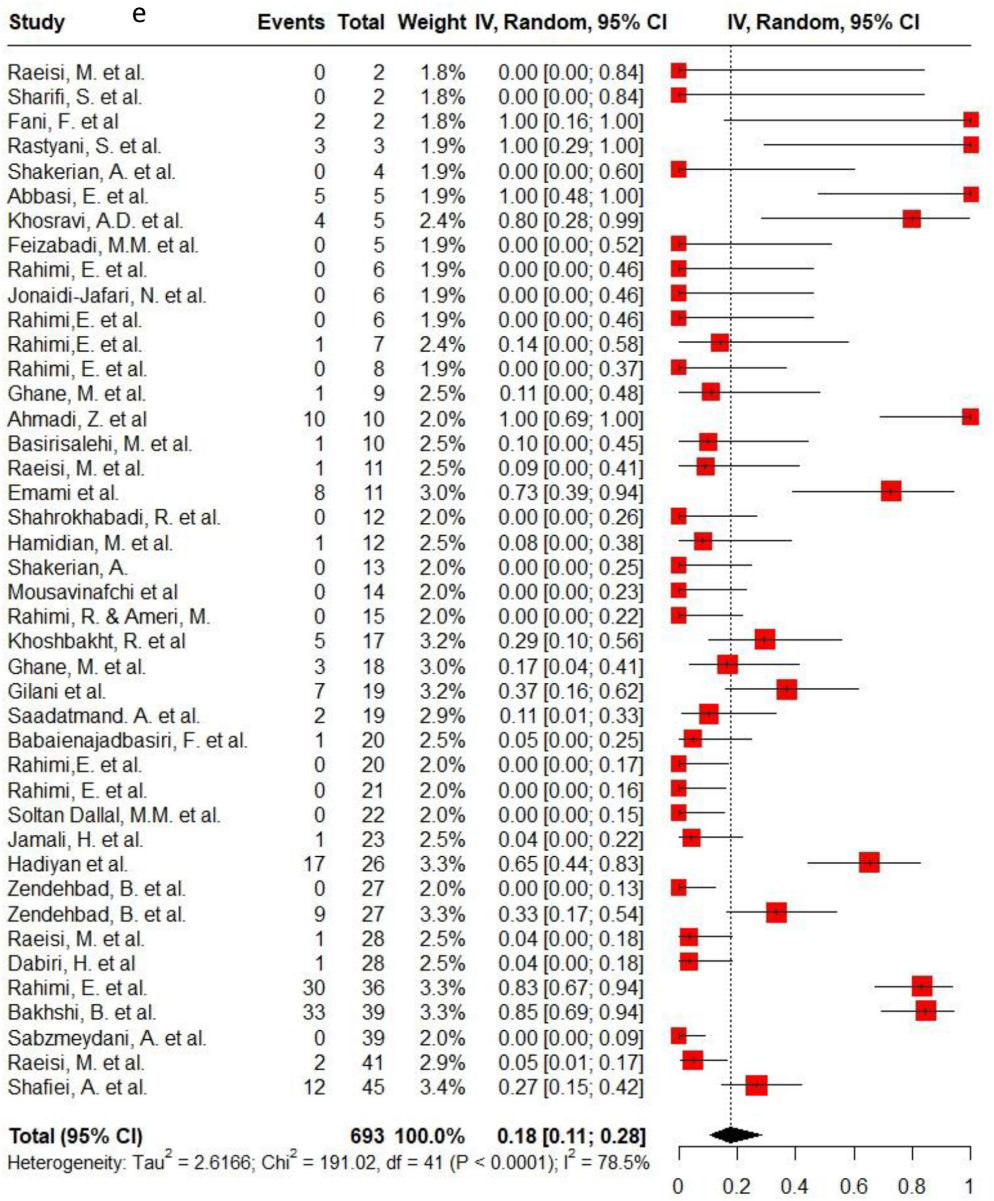

**Figure:**
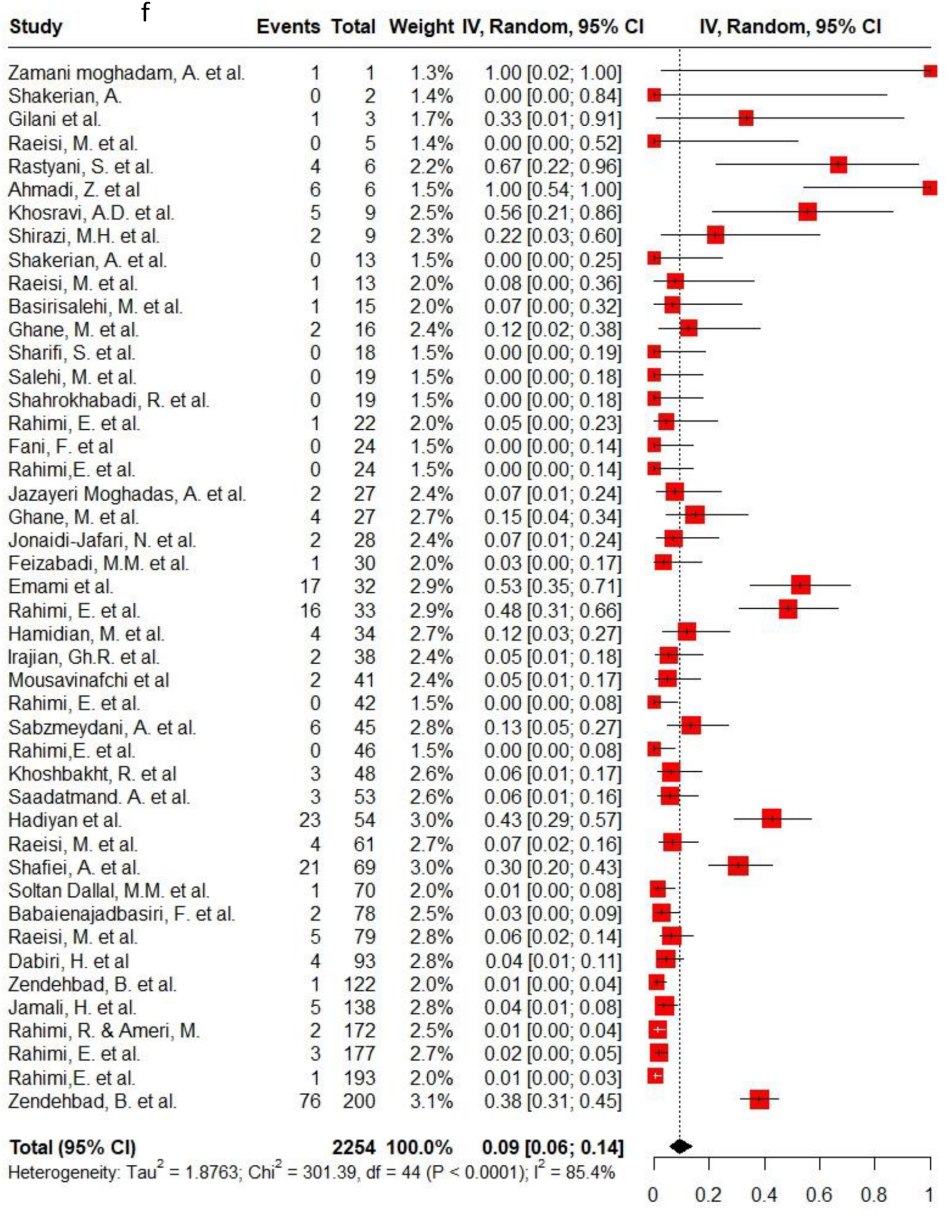

**Figure:**
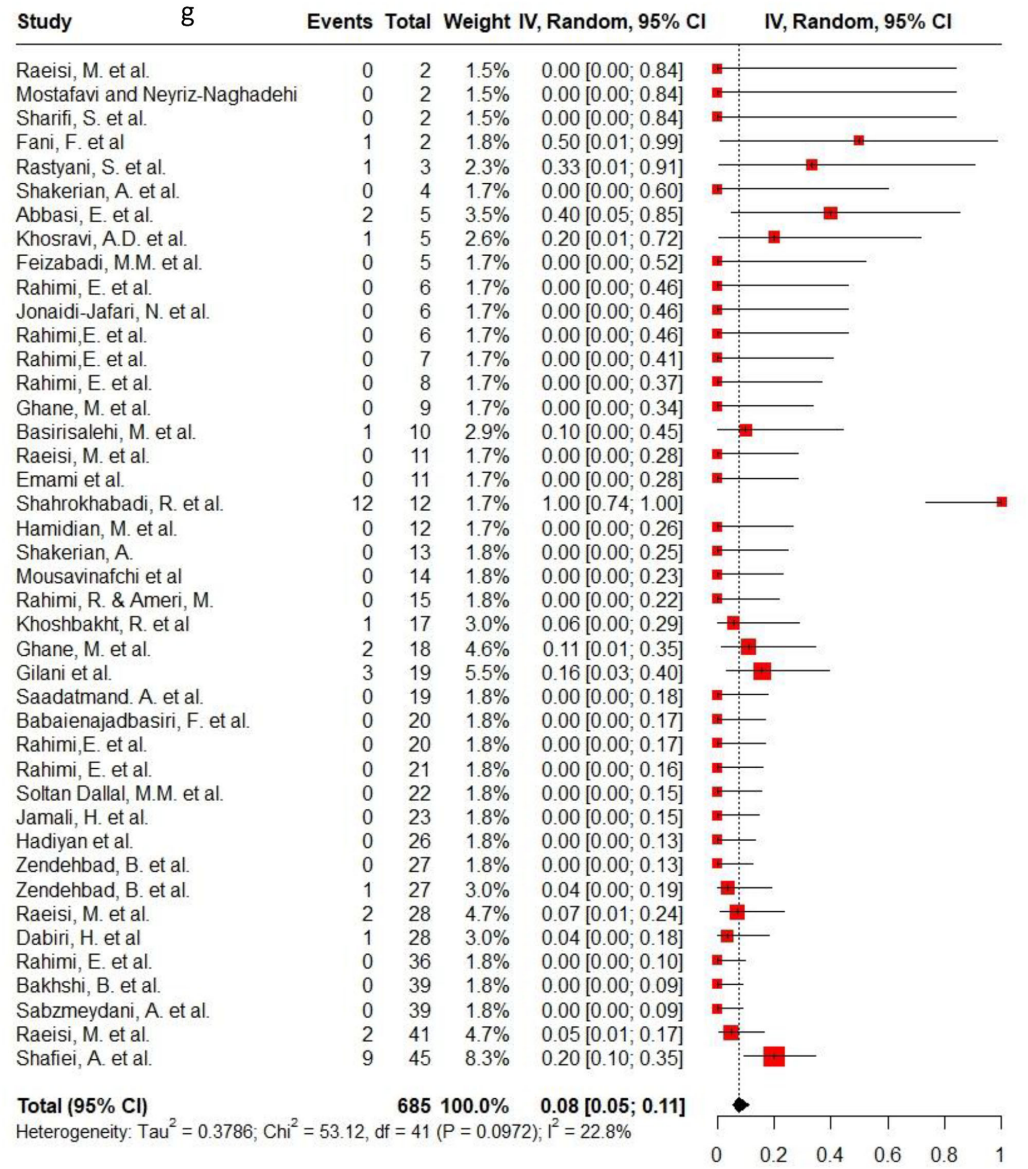

**Figure:**
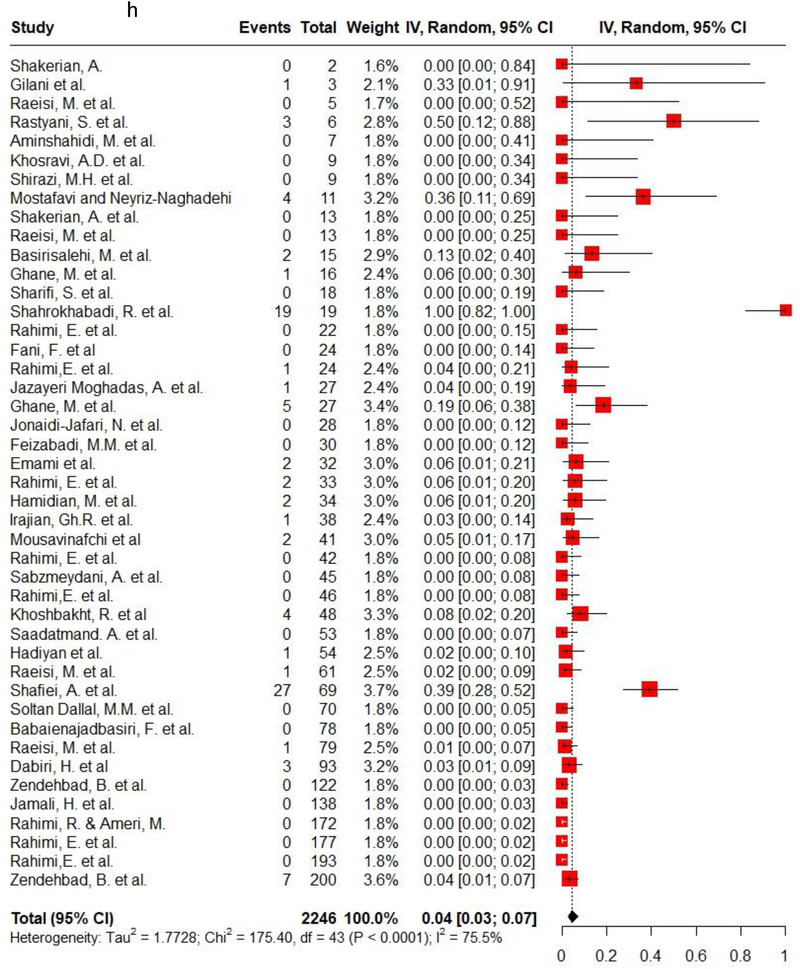

**Figure:**
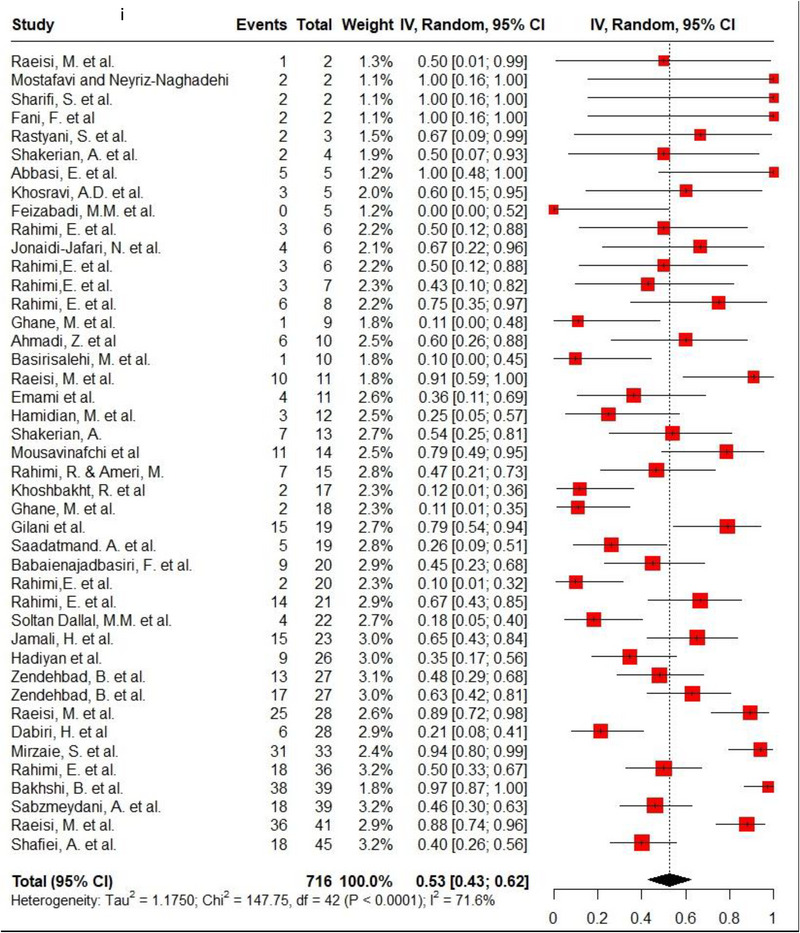

**Figure:**
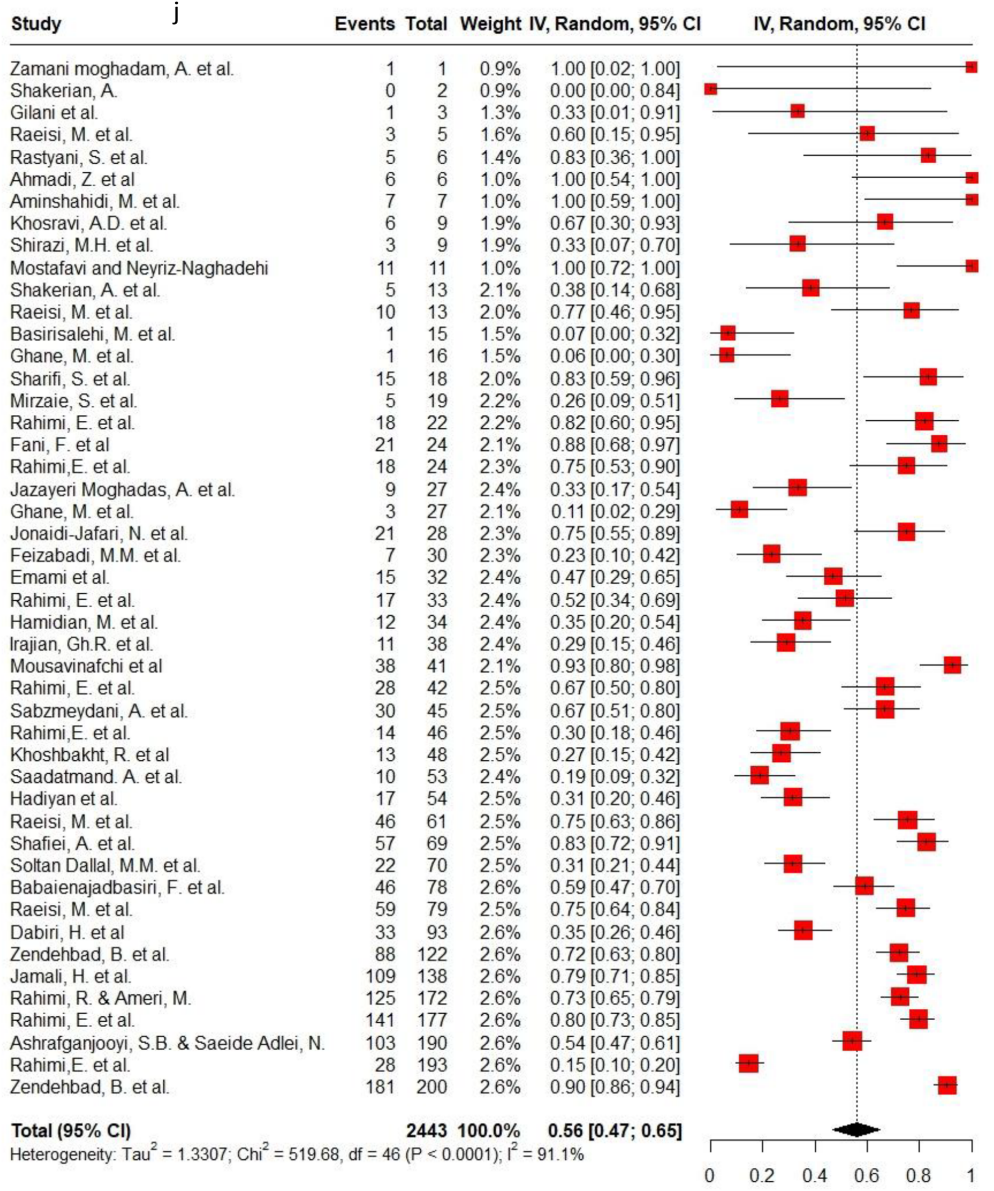

### 3.3 Pooled Prevalence of ARG in *Campylobacter* spp. in Human

The most prevalent ARG in *Campylobacter* spp. was *tetO* (0.73; 95% CI: 0.46–0.90; *I* ^2^: 90%; seven studies) and *cmeB* (0.48; 95% CI: 0.32–0.63; *I* ^2^:78; four studies) and *bla*_OXA61_ (0.42; 95% CI: 0.06–0.9; *I* ^2^: 91%; six studies). The prevalence of *gyrA6* (0.32; 95% CI: 0.22–0.44), *qnrs* (0.31; 95% CI: 0.20–0.43), *A23srRNA* (0.3; 95% CI: 0.19–0.41), *gyrA4* (0.29; 95% CI: 0.19–0.40) and *gyrA5* (0.18; 95% CI: 0.09–0.28) genes was investigated in one study and was considerably high, whereas the lowest prevalence was observed for *aphA* (0.2; 95% CI: 0–0.7; one study), *tetA* (0.06; 95% CI: 0.03–0.10; two studies) and *tetB* and *tetS* (0.0%; 95% CI: 0.0–0.1; two studies).

## 4 Discussion

Foodborne campylobacteriosis was reported in 2 out of 102 reported outbreaks in Iran (Soltan Dallal et al. 2017). The emergence of antimicrobial‐resistant pathogens is an ongoing global concern in both human and veterinary medicine. The indiscriminate and excessive utilization of the antimicrobial in veterinary medication, especially in food animals and agricultural sectors, exacerbates the issue as they can be transmitted to humans through the food chain (Bennani et al. 2020). In this respect, the development of AMR in *Campylobacter* spp. has become a serious threat to public health (Asuming‐Bediako et al. 2019; Jamali et al. 2015; Khademi and Sahebkar 2020). *Campylobacter* spp. is one of the most commonly involved pathogens in human gastroenteritis around the world (Asuming‐Bediako et al. 2019; Azizian et al. 2019; Modirrousta et al. 2016).

In the ongoing review, we present the AMR proportions in *Campylobacter* spp. in Iran, based on a systematic review of published articles from the country. Our findings demonstrated that the majority of *Campylobacter* spp. isolates from Iran showed high resistance rates to fluoroquinolones (including ofloxacin, nalidixic acid and ciprofloxacin), beta‐lactams (including cephalothin, cephalexin, ceftazidime and carbenicillin), sulfonamides (including potentiated sulfonamide trimethoprim‐sulfamethoxazole), florfenicol and tetracycline, which are among the critically and highly important antimicrobials for treatment of human infections. A recent study in intensive care units of a hospital in Iran showed that AMR against trimethoprim‐sulfamethoxazole (61.7%), ciprofloxacin (51.3%), imipenem (50.0%) and ampicillin (49.6%) was the most common observed finding (Salarvand et al. 2023). Our results from Iran were practically identical to those recently reported from different countries (Abdallah et al. 2022; de Vries et al. 2018; Hakkinen et al. 2007; Igwaran and Okoh 2020; Little et al. 2008; Rangaraju et al. 2022; Sadeghi et al. 2022). Similar to our results, rising resistance proportions of *Campylobacter* isolates to fluoroquinolones have also been documented in the United States and the United Kingdom, where a steadily increasing trend has been observed (Luangtongkum et al. 2006; Tang et al. 2017; Veltcheva et al. 2022). Additionally, in Southern Europe, the prevalence of fluoroquinolone‐resistant *Campylobacter* expanded altogether from 1997 to 2001. Erythromycin resistance has remained low and consistent (2%) (Gupta et al. 2004), which was lower than the present study (Gao et al. 2023).

Chatre et al. (2010) documented the AMR of *Campylobacter* isolated from cattle between 2002 and 2006 in France. They discovered that while ampicillin, erythromycin and gentamicin showed low (<1%) resistance, most isolates (>50%) had high tetracycline and nalidixic acid resistance. Resistance to fluoroquinolone increased from 29.7% to 70.4% between 2002 and 2006. Moreover, Chen et al. (2010) revealed that 98% of *Campylobacter* isolates in poultry from China were also resistant to quinolones (nalidixic acid, ciprofloxacin and enrofloxacin) and tetracyclines (tetracycline and doxycycline). On the other hand, our results established that *Campylobacter* spp. were vulnerable or appeared to have low resistance proportions to aminoglycosides (including spectinomycin (0.04), gentamicin (0.04), neomycin (0.08) and amikacin (0.08)). Similar findings have been detailed in previous studies conducted in other nations (Chatre et al. 2010; Gupta et al. 2004). Similar to our observations from Iran, resistance to macrolides (including azithromycin (0.17) and erythromycin (0.12)), Rangaraju et al. (2022) observed that the resistance rate to macrolides (61.53%) in poultry settings in India was among the highest AMR rates. More than 90% of *Campylobacter* strains (761 *C. jejuni* and 130 *C. coli*) had resistance to ciprofloxacin, nalidixic acid and tetracycline (Gao et al. 2023). Furthermore, Chen et al. (2010) reported the isolation rate and AMR profile of *Campylobacter* isolated from broilers in China. They stated that *Campylobacter* isolates exhibited a moderate‐to‐high (>5%–50%) rate of resistance to macrolides and gentamicin, which is similar to our findings. Fluoroquinolones are a medication of choice for treating *Campylobacter* infection. These results reflect that the fluoroquinolone resistance prevalence rates of *Campylobacter* from Iran are similar to those reported from Asia and Africa (>80%), the United States and Canada (19%–47%) and European countries (17%–99%) (Luangtongkum et al. 2009). This is consistent with the World Health Organization report on fluoroquinolone resistance in *Campylobacter* species worldwide (Luangtongkum et al. 2009). Different mechanisms are attributed to the occurrence of chromosomally mediated quinolone resistance in *Campylobacter* species, such as *CmeABC*, efflux pumps and single‐point mutations in DNA *gyrase A* (*GyrA*), such as the *C257* T mutation, which is the most common mutation (Luangtongkum et al. 2009). The cases of fluoroquinolone‐resistant *Campylobacter* in consumers of poultry meat were reported by the FDA (Dafale et al. 2020).

Macrolides are another drug of choice used in *Campylobacter* infection therapy. Our results showed resistance in *Campylobacter* species to macrolides (erythromycin 12% and azithromycin 17%). The prevalence of erythromycin resistance in Iran (0.12) was higher than that in Canada (Narvaez‐Bravo et al. 2017), the Czech Republic (Bardon et al. 2009), Australia (Miflin et al. 2007), and Turkey (Ozbey and Tasdemir 2014), and lower than that in China (Han et al. 2016), Malaysia (Premarathne et al. 2017) and South Africa (Shobo et al. 2016). Three important mechanisms related to macrolide resistance in *Campylobacter* species include target modifications of *gyrA* and *23S rRNA* chromosomal genes through point mutations, ribosomal proteins (i.e., L4 and L22) and the *CmeABC* efflux pump (Luangtongkum et al. 2009; Sharifi et al. 2021). Alternative antibiotics for the treatment of campylobacteriosis are tetracyclines and gentamicin. The mode of action attributed to tetracycline resistance in *Campylobacter* species is the modification of ribosomal protection proteins (*tet*O and *tet*S), efflux protein genes (*tet*A and *tet*B) and the *CmeABC* efflux pump (Abdi‐Hachesoo et al. 2014; Luangtongkum et al. 2009; Sharifi et al. 2021). Moreover, aminoglycoside‐modifying enzymes play a critical role in aminoglycoside resistance in *Campylobacter* species (Luangtongkum et al. 2009). On the basis of our findings, tetracycline antibiotic resistance in *Campylobacter* was higher than gentamicin. Similar outcomes were reported in studies previously conducted in Turkey (Ozbey and Tasdemir 2014), South Korea (Ozbey and Tasdemir 2014), Poland (Maćkiw et al. 2012), Italy (Pezzotti et al. 2003) and Africa (de Vries et al. 2018). Similar to our findings, studies conducted in Turkey have reported notable levels of resistance (>50%) to tetracycline (Ozbey and Tasdemir 2014), China (Han et al. 2016), Malaysia (Premarathne et al. 2017), Italy (Pezzotti et al. 2003), Poland (Maćkiw et al. 2012), Canada (Narvaez‐Bravo et al. 2017), South Africa (Shobo et al. 2016) and South Korea (Wei et al. 2014). Our study also showed that antibiotic resistance proportions to beta‐lactam antibiotics, including cefixime, cephalothin, ceftriaxone, cephalexin and ceftazidime, were high. However, the resistance proportions to imipenem and meropenem were low. The production of beta‐lactamase enzymes and intrinsic resistance are the two main mechanisms by which *Campylobacter* species are resistant to alkaline antibiotics (Aminshahidi et al. 2017; Luangtongkum et al. 2009).

In most of the antibiotics, a higher resistance was seen in human isolates than others. Food isolates had higher resistance to doxycycline, neomycin, imipenem, azithromycin, colistin, ciprofloxacin, trimethoprim‐sulfamethoxazole and tetracycline. It was reported 30%–90% of administered antibiotics in animals are defecated unchanged, which, when applied as fertilizer, creates a major reservoir of antibiotic residues, antibiotic‐resistant bacteria and ARGs (Enshaie et al. 2025). These residues are able to pollute soil and water via run‐off or discharge, promoting the persistence and proliferation of antibiotic‐resistant bacteria and ARGs, which can be transferred across species, including to humans. Soil bacteria can remain as long‐term reservoirs of ARGs, which can be transmitted to pathogens, creating a cycle from the environment to humans via crops or water (Enshaie et al. 2025). Humans can take up the resistant bacteria via direct contact with animals, consumption of contaminated animal products such as meat or milk, and environmental sources like water contaminated with animal waste (Al‐Nasser et al. 2020).

According to the findings of the present investigation, the proportion of erythromycin and gentamycin resistance was higher in C. *coli* than *C. jejuni*, whereas the resistance to ciprofloxacin and tetracycline was higher in *C. jejuni* than *C. coli* isolates. Erythromycin resistance of *C. coli* (59.23%) was higher than *C. jejuni* (2.50%) in clinical isolates in Shanghai, China (Gao et al. 2023). The authors who investigated the prevalence and antibiotic resistance rates of *Campylobacter* in United States dairy cattle concluded that *C. coli* strains showed higher resistance to antimicrobials than *C. jejuni* (Englen et al. 2007). Moreover, *C. coli* isolates had higher resistance to antimicrobials than *C. jejuni* from diarrhoeal patients and chickens in Botswana (de Vries et al. 2018). Eryildiz et al. (2020) reported the higher resistance to antimicrobials in *C. jejuni* (Eryildiz et al. 2020). *C. jejuni* and *C. coli* were resistant to ciprofloxacin, tetracycline, florfenicol and nalidixic acid (5.39%), and azithromycin, ciprofloxacin, erythromycin, gentamicin, tetracycline, clindamycin and nalidixic acid (28.46%), respectively (Gao et al. 2023).

AMR is increasingly conceptualized as a critical environmental concern, transcending its conventional association with clinical and veterinary domains. The natural environment operates as a fundamental reservoir, conduit and catalyst in the emergence, genetic evolution and dissemination of antimicrobial‐resistant bacteria and ARGs. Within the framework of the One Health paradigm—which underscores the intrinsic interdependence of human, animal and environmental health—the environmental dimension is now regarded as indispensable to understanding the complex epidemiological and ecological dynamics underpinning AMR (Andersson and Hughes 2014).

On the basis of our findings, the most prevalent ARG in *Campylobacter spp*. was *tetO*, followed by *cmeB, OXA61, gyrA6*, *qnrs*, *A23srRNA*, *gyrA4* and *gyrA5* genes. The least prevalent AMR genes were *tetB* and *tetS*, *aphA* and total *tetA*. Resistance mechanisms observed in *Campylobacter* include horizontal gene transfer between different *Campylobacter* species, such as that observed with tetracycline (*tetO, A, B, S*), erythromycin (*aphA*), chloramphenicol, neomycin, kanamycin and streptomycin genes, as well as obtaining the resistance genes from other bacterial species, such as the kanamycin (*aphA‐3*), streptomycin (*aadA*) and streptomycin/spectinomycin (*aadE*) resistance genes obtained from Gram‐positive cocci (Aarestrup and Wegener 1999). The *tet*(O) and *23SrRNA* genes were recorded in 54.55% and 50% of tetracycline‐ and macrolide‐resistant isolates, respectively (Rangaraju et al. 2022). Analysis of environmental samples, including sewage, seawater, sediment and aerosol, documented widespread ARGs and also identified the overlapping of ARGs (Berendonk et al. 2015). Regarding the One Health approach, none of the studies in Iran used this approach to interlink these sectors in the assessment of AMR in *Campylobacter*. ARGs just have been studied in a few studies in human and food samples.

### 4.1 Limitations

One of the limitations of this study is the report of different antimicrobial agents in different samples, and also different food samples were studied in different research studies. Moreover, other limitations were the wide timeframe of included studies and lack of recent studies and also the low number of studies reporting ARGs. In addition, the findings of the current study must be interpreted cautiously due to high heterogeneity. Results of this study showed that, as the antibiotic resistance rates were different in different sectors and some of them had no considerable difference, control of AMR must be considered in all sectors.

## 5 Conclusion

Our meta‐analysis results showed that *Campylobacter* species isolated from humans, animals and food in Iran have a high proportion of resistance to different antibiotics. To reduce the resistance rate of pathogens, different strategies such as frequent drug resistance monitoring, reduced and optimized use of antimicrobials in all sectors, education and raising awareness towards AMR have been suggested. Moreover, good agricultural and manufacturing practices and hazard analysis of critical control points at every stage of the food chain are recommended to decrease the risk of development and spread of antibiotic resistance. In addition, the investigation of ARGs in different sections, especially environmental sources, is suggested.

## Author Contributions

Fatemeh Salmani, Sara Mohamadi, Parisa Sadighara and Tayebeh Zeinali conceptualized the study. Tayebeh Zeinali and Parisa Sadighara performed the search and extract the data. Sara Mohamadi and Tayebeh Zeinali evaluate the studies. Fatemeh Salmani performed the analysis. Taurai Tasara critically revised the article. All authors coorporate in the writing of the draft. All authors read and approved the final manuscript.

## Funding

The authors have nothing to report.

## Ethics Statement

The study was approved by ethical committee of Birjand University of Medical Sciences.

## Conflicts of Interest

The authors declare no conflicts of interest.

## Data Availability

Data are available from the corresponding author on reasonable request.
